# Rewiring the mitochondrial NAD^+^–CoQ axis using metabolic nanomedicine for acute kidney injury therapy

**DOI:** 10.1016/j.mtbio.2026.103479

**Published:** 2026-07-22

**Authors:** Huaze Ding, Xinhui Huang, Peng Hu, Xiaoyan Zhang, Yulin Wang, Baiwei Mao, Jianxin Ye, Dianwen Song, Chong Zhang, Changping Wang

**Affiliations:** a Department of Orthopedics, Shanghai General Hospital, Shanghai Jiao Tong University School of Medicine, Shanghai, 200080, China; b Department of Nephrology, Xin Hua Hospital Affiliated to Shanghai Jiao Tong University School of Medicine, Shanghai, 200092, China; c Department of Nephrology, Zhongshan Hospital, Fudan University, Shanghai, 200032, China; d Department of Nephrology, Shanghai General Hospital, Shanghai Jiao Tong University School of Medicine, Shanghai, 200080, China; e Division of Spine, Department of Orthopedic Surgery, Shenzhen People's Hospital, Shenzhen, 518020, China

## Abstract

Acute kidney injury (AKI) represents a critical medical condition with high mortality and no effective pharmacotherapy. Mitochondrial dysfunction in proximal tubular epithelial cells (PTECs), characterized by NAD^+^ depletion, CoQ oxidation, and reactive oxygen species (ROS) overproduction, is a central driver of tubular damage and maladaptive repair. As central metabolic molecules, NAD^+^ and CoQ can effectively ameliorate acute kidney injury by alleviating mitochondrial oxidative stress. However, the bioavailability of NAD^+^ is limited by its short half-life, instability, and poor membrane permeability, whereas CoQ, despite being lipophilic, lacks specific mitochondrial targeting, preventing maximal therapeutic efficacy. Here, we report carrier-free NAD^+^/MitoQH_2_ nanoparticles (NM NPs) with ultrasmall size and ROS-responsive properties. NM NPs are designed to pass through the glomerular filtration barrier and preferentially accumulate in injured renal tubules, where they may replenish the NAD^+^ pool and provide a pre-reduced, mitochondria-targeted ubiquinol to support the CoQ axis under oxidative stress. Through renal accumulation and ROS-responsive release, NM NPs showed therapeutic efficacy in hypoxia/reoxygenation- and cisplatin-induced AKI models, as evidenced by reduced ROS accumulation, improved mitochondrial oxidative phosphorylation, enhanced autophagy, and attenuated ferroptosis. This work presents a metabolic nanomedicine strategy based on the co-assembly of bioactive molecules to modulate the mitochondrial NAD^+^–CoQ axis, providing a promising platform for AKI therapy and potentially other mitochondria-associated diseases.

## Graphical abstract

**Figure:**
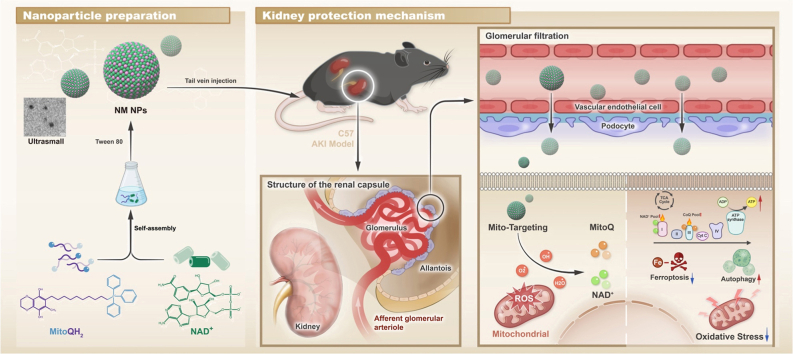

## 1 Introduction

Acute kidney injury (AKI) is defined by a rapid loss of renal function with accumulation of nitrogenous waste and electrolyte imbalance [1,2].It carries a high risk of progression to chronic kidney disease (CKD) and end-stage renal disease [3]. Despite decades of research, clinical management of AKI remains largely supportive [4]. Current practice focuses on hemodynamic optimization, avoidance of nephrotoxins and renal replacement therapy in severe cases, and no approved drug can modify the disease course [5,6]. Among the diverse causes of AKI, renal ischemia-reperfusion (IR) and cisplatin nephrotoxicity are two major clinical settings [7]. Both converge on proximal tubular epithelial cells (PTECs) as primary targets, which have very high metabolic demand and a dense mitochondrial network to sustain solute reabsorption [8,9]. When mitochondrial function fails, oxidative phosphorylation declines, reactive oxygen species (ROS) increase and cellular energy collapses [10,11]. Mitochondrial dysfunction has therefore become a central driver of tubular injury and incomplete repair in AKI [12]. These features highlight an urgent need for therapies that protect and restore mitochondrial function in the injured kidney.

A key feature of mitochondrial stress in AKI is marked depletion of nicotinamide adenine dinucleotide (NAD^+^) [13]. NAD^+^ is both a redox cofactor and a substrate for sirtuins and other NAD^+^-consuming enzymes [14].Several studies show that ischemia, oxidative stress and DNA damage activate NAD^+^-consuming pathways such as PARP1, while NAD^+^ biosynthesis is suppressed at the same time [[15], [16], [17]]. As a result, the cellular NAD^+^ pool falls rapidly and remains low [15]. Loss of NAD^+^ impairs mitochondrial dehydrogenases and NAD^+^-dependent regulators such as Sirt3, disrupts the electron transport chain (ETC), reduces ATP production and increases susceptibility to apoptosis, ferroptosis and inflammatory signaling [18,19]. Pharmacological strategies that raise NAD^+^ levels using precursors such as nicotinamide riboside, nicotinamide mononucleotide or nicotinamide have shown protective effects in AKI and in limiting fibrotic progression in preclinical models [[20], [21], [22]]. However, systemic precursor therapy often requires high and repeated doses because of non-specific uptake in peripheral tissues and variable conversion to NAD^+^, and may cause off-target inflammatory effects [23]. Direct delivery of NAD^+^ is even more difficult. As a highly hydrophilic and negatively charged molecule, NAD^+^ crosses membranes poorly, is rapidly cleared and shows limited renal and mitochondrial accumulation when administered in free form [[24], [25], [26]]. Nanoparticle-based delivery of NAD^+^ or its precursors can improve stability and organ distribution, but many systems rely on inorganic cores or complex multi-component architectures with unresolved issues in subcellular targeting, long-term safety and manufacturability [27,28]. Efficient and safe replenishment of the intrarenal and mitochondrial NAD^+^ pool in AKI therefore remains a major challenge.

In parallel, mitochondria-targeted antioxidants such as triphenylphosphonium (TPP^+^)-conjugated ubiquinone (MitoQ) have been developed to reduce mitochondrial ROS and stabilize membranes [29]. The classical paradigm assumes that oxidized MitoQ reaches the inner mitochondrial membrane and is enzymatically reduced to its active ubiquinol form (MitoQH_2_) by complex II (succinate dehydrogenase) or NAD(P)H:quinone oxidoreductase 1 (NQO1) [30]. MitoQH_2_ can then help replenish the CoQ pool and prevent lipid peroxidation [31]. This activation pathway depends on the integrity of enzymes that are selectively damaged in AKI. In IR, succinate accumulates and complex II function is disturbed, while in cisplatin nephrotoxicity complex II and NQO1/Nrf2 pathways are frequently inhibited or downregulated [32]. Under these conditions, oxidized MitoQ is converted to MitoQH_2_ less efficiently and may instead build up as a cationic quinone in the inner membrane [33]. High intramitochondrial TPP^+^ loads and redox-active quinone or semiquinone species raise concerns about membrane depolarization and pro-oxidant effects when electron flow is already impaired. From a bioenergetic perspective, effective mitochondrial protection in AKI requires both restoration of electron donors and stabilization of the electron carrier pool [32]. NAD^+^ depletion disconnects upstream dehydrogenases from the ETC, whereas oxidation and depletion of CoQ interrupt electron transfer along the inner membrane and promote lipid peroxidation and ferroptotic injury [34]. Supplementing NAD^+^ or its precursors can partly restore dehydrogenase activity and Sirt3 signaling, but if the CoQ pool remains damaged or oxidized, electron flow through the ETC stays inefficient and mitochondria remain vulnerable to ROS [35]. Delivering a mitochondria-targeted ubiquinol alone can strengthen membrane antioxidant defenses and limit lipid peroxidation, but cannot resolve an upstream NAD^+^ bottleneck that restricts substrate oxidation and ATP generation. In other words, supplying fuel without a functional carrier system, or stabilizing the wires without restoring fuel, is insufficient in AKI. We therefore hypothesized that simultaneous replenishment of the NAD^+^ pool and provision of a pre-reduced, mitochondria-targeted ubiquinol could more effectively restore mitochondrial electron flow in damaged renal tubules. NAD^+^ supports upstream substrate oxidation and redox metabolism, whereas MitoQH_2_ stabilizes the CoQ-dependent electron carrier and antioxidant buffering system at the inner mitochondrial membrane. Their co-delivery may therefore reconnect electron-donor supply with electron-transfer capacity, reducing mitochondrial ROS and lipid peroxidation while modulating ferroptosis- and autophagy-related stress responses.

**Scheme 1:**
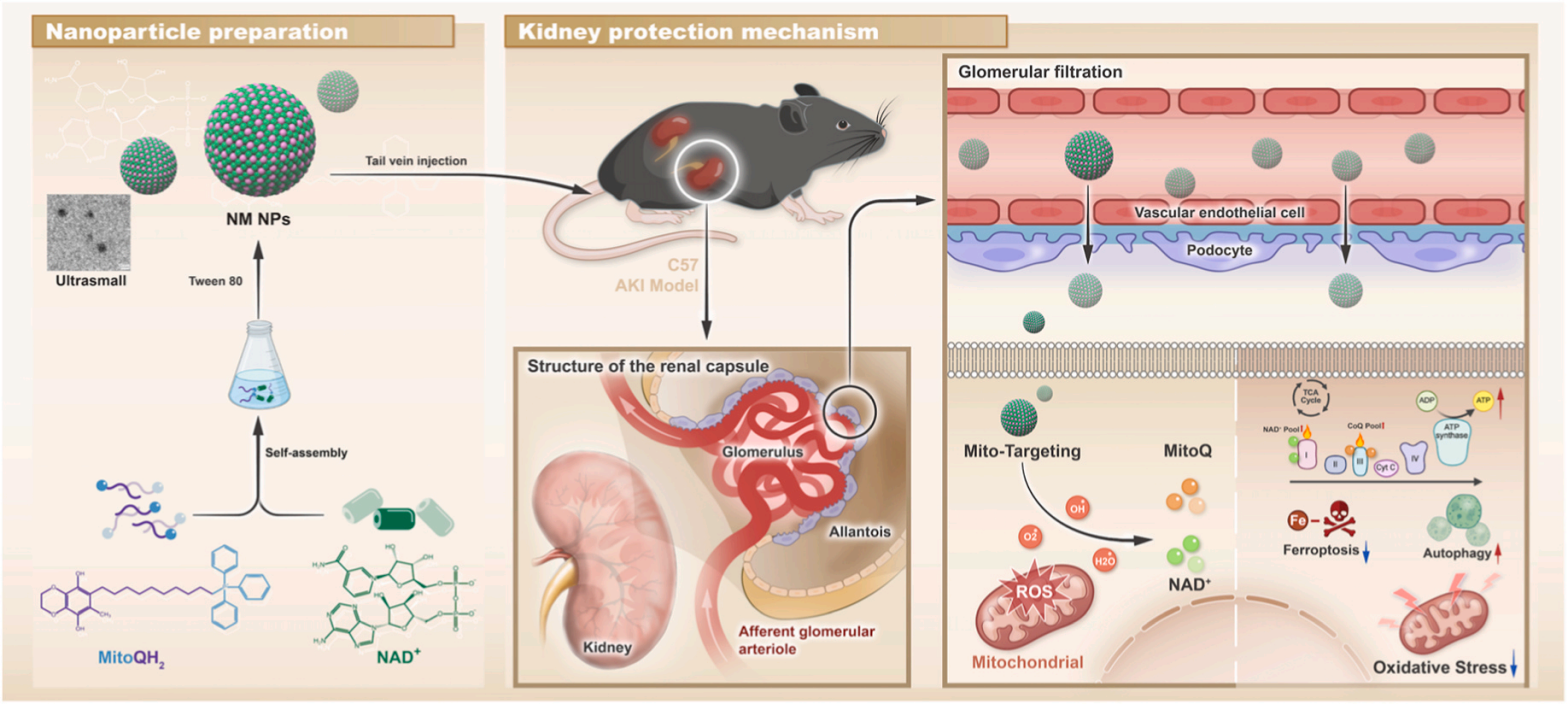
**Ultrasmall NAD^+^/MitoQH_2_ Nanoparticles Enable Renal Mitochondrial Protection Against Acute Kidney Injury.** NM NPs are generated by supramolecular co-assembly of NAD^+^ and MitoQH_2_, into well-defined, ultrasmall (∼10 nm) nanoparticles with intrinsic ROS-responsive properties. Following systemic administration, NM NPs reach the kidney and, due to their ultrasmall size, undergo glomerular filtration to enter the renal parenchyma. In injured renal cells, NM NPs deliver and release NAD^+^/MitoQ to mitochondria, reinforcing the NAD^+^–CoQ axis to enhance electron transport chain flux, thereby suppressing mitochondrial ROS and oxidative stress, restoring mitochondrial function, enhancing autophagy, and attenuating ferroptosis to confer renoprotection.

To implement this concept, the delivery system must satisfy several additional constraints that arise from renal physiology and redox pharmacology. The glomerular filtration barrier imposes a strict size cutoff [36]. Ultrasmall nanoparticles with diameters below about 10–15 nm can pass through this barrier, enter the tubular lumen and concentrate at the PTEC brush border, which enables passive yet selective enrichment at the main sites of injury [37]. To overcome the poor membrane permeability and short circulation time of NAD^+^, the carrier should promote cellular uptake and subcellular localization without using bulky or strongly immunogenic scaffolds. The pre-reduced ubiquinol must be protected from premature oxidation and redox cycling in the bloodstream, yet be able to disassemble and release its cargo in the ROS-rich microenvironment of injured tubules. MitoQH_2_, the reduced form of MitoQ, combines a TPP^+^ motif that drives accumulation at mitochondria with a ubiquinol headgroup that participates directly in antioxidant and electron transport processes. Embedding NAD^+^ within ultrasmall MitoQH_2_-based assemblies offers a way to use TPP^+^-driven mitochondrial targeting to help chaperone NAD^+^ across cellular barriers, to co-localize NAD^+^ and a pre-reduced CoQ analogue near the ETC and to exploit elevated ROS as a trigger for controlled disassembly and cargo release. Here, we report ultrasmall, carrier-free NAD^+^/MitoQH_2_ nanoparticles (NM NPs) that satisfy these criteria and protect renal mitochondria in both hypoxia- and cisplatin-induced AKI. NAD^+^ and MitoQH_2_ self-assemble into ∼10 nm spherical particles with defined composition, good colloidal stability and ROS-responsive disassembly (Scheme 1). After systemic administration, NM NPs are filtered by the glomerulus, selectively accumulate in injured kidneys and are internalized by PTECs, where the TPP^+^ motif promotes mitochondrial localization and elevates intramitochondrial NAD^+^ and CoQ, thereby reducing ROS and restoring oxidative phosphorylation. In both cell and mouse models of IR- and cisplatin-induced AKI, NM NPs attenuate tubular injury, improve renal function, suppress ferroptosis and enhance autophagy and mitophagy. Thus, NM NPs provide a minimalist platform for organ- and organelle-specific therapy in AKI and exemplify NAD^+^–CoQ axis rewiring through co-delivery of a metabolic cofactor and a pre-reduced mitochondria-targeted ubiquinol.

## 2 Results and discussion

### 2.1 Design and characterization of ultrasmall ROS-responsive NM NPs

**Fig. 1:**
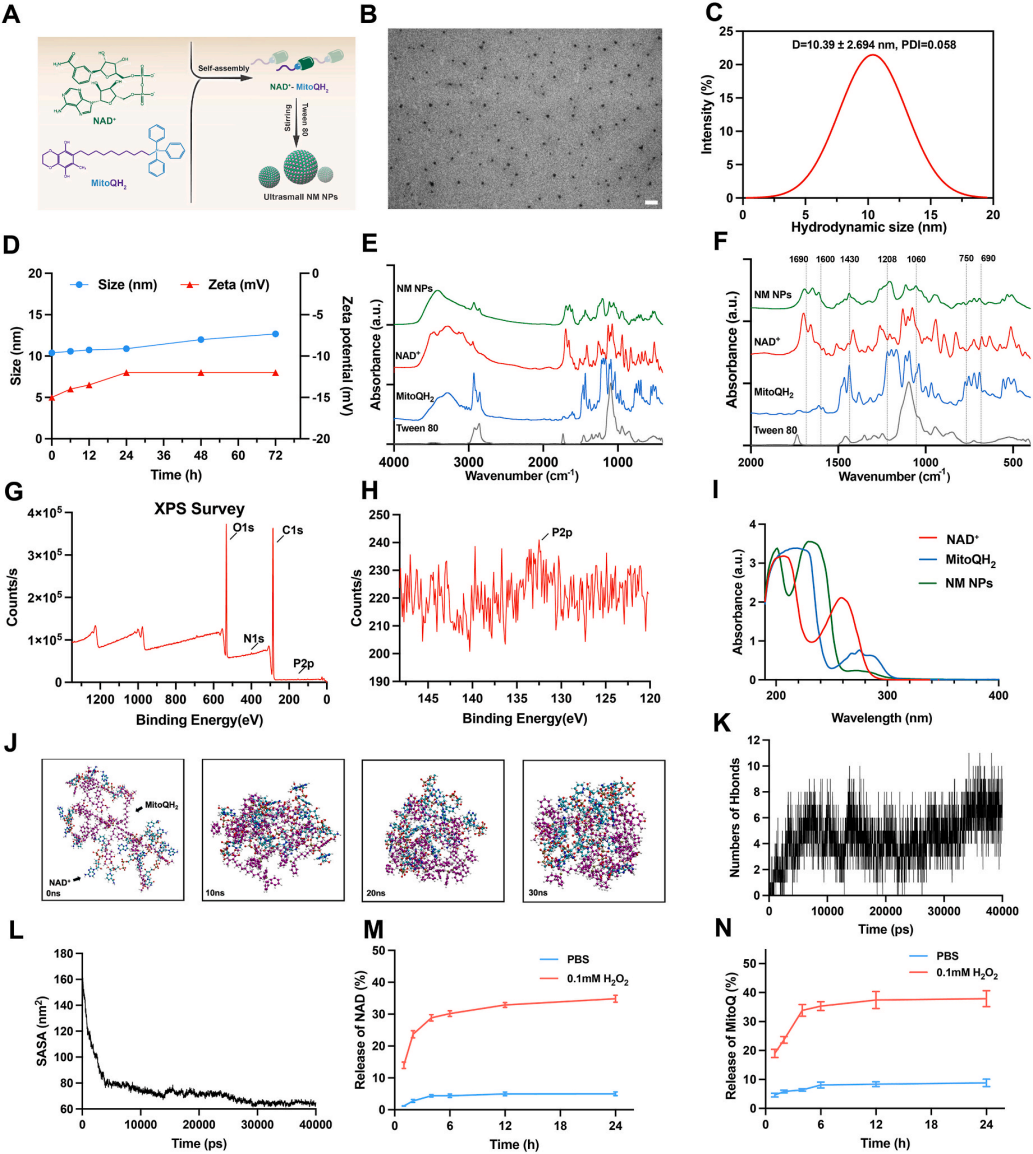
**Preparation and characterization of NM NPs.** (A) Schematic illustration showing the preparation process of ultrasmall NM NPs. (B) TEM image of NM NPs. Scale bar = 10 nm. (C) DLS analysis of NM NPs. (D) Time-dependent particle size and zeta potential of NM NPs at 4 °C. (E-F) FTIR spectra of NM NPs, NAD^+^, MitoQH_2_, and Tween 80. (G-H) XPS survey and P 2p spectra of NM NPs. (I) UV–vis absorption spectra of NAD^+^, MitoQH_2_, and NM NPs. (J) Representative MD snapshots of NM NP formation at different time points. (K) Time evolution of hydrogen bonds during the assembly process of NM NPs. (L) Solvent-accessible surface area of NM NPs over simulation time. (M − N) Release profiles of NAD^+^ and MitoQ from NM NPs in PBS and 0.1 mM H_2_O_2_, n = 3 per group. Data are mean ± SD.

To address the unmet need for targeted and responsive nanotherapeutics in AKI, we developed a self-assembled nanomedicine through the co-assembly of NAD^+^ and the mitochondrially-targeted antioxidant MitoQH_2_. NM NPs were synthesized under mild conditions (4 °C) by co-dissolving NAD^+^ and MitoQH_2_ at an equal mass ratio in aqueous solution containing the non-ionic surfactant Tween 80, followed by vigorous stirring overnight (Fig. 1A). The assembly is driven by a synergistic combination of π–π stacking, hydrophobic interactions, and electrostatic pairing, resulting in the formation of stable, monodisperse nanostructures. Transmission electron microscopy (TEM) imaging revealed that the nanoparticles exhibited a uniform spherical morphology with an average diameter of approximately 10 nm (Fig. 1B). This ultrasmall size was further corroborated by dynamic light scattering (DLS) analysis, which showed a narrow hydrodynamic size distribution centered at 10.39 ± 2.694 nm with a low PDI value of 0.058 (Fig. 1C). Such ultrasmall nanoparticles exhibit excellent stability. The nanoparticles demonstrated excellent colloidal stability over 72 h in aqueous medium, with only a minimal change in particle size, zeta potential and PDI value (Fig. 1D and S1-2), which is critical for *in vivo* application. To determine the individual proportions of NAD^+^ and MitoQH_2_ in NM NPs, the encapsulation efficiency and loading content of NAD^+^ and MitoQH_2_ were quantitatively analyzed by LC-MS. The encapsulation efficiencies of NAD^+^ and MitoQH_2_ were 87.39 ± 1.61% and 69.31 ± 1.38%, respectively (Table S1). Further analysis of lyophilized NM NPs showed that NAD^+^ and MitoQH_2_ accounted for 52.48 ± 1.18% and 41.54 ± 0.85% of the total nanoparticle mass, respectively (Table S2). These results quantitatively confirm the successful incorporation of both NAD^+^ and MitoQH_2_ into NM NPs.

The FTIR spectra were analyzed to verify the assembly of NAD^+^ and MitoQH_2_ and to assess the possible retention of Tween 80 after purification. In the full-range spectra (Fig. 1E), the broad band at 3200–3500 cm^−1^ was attributed to O–H/N–H stretching vibrations from NAD^+^ and MitoQH_2_, suggesting hydrogen-bonding interactions during nanoparticle formation. The bands at 2920–2850 cm^−1^ corresponded to aliphatic C–H stretching, mainly from the alkyl chain of MitoQH_2_. In the fingerprint region (Fig. 1F), the peaks at approximately 1690 and 1600 cm^−1^ were assigned to C=O stretching and aromatic C=C/C=N skeletal vibrations, respectively, confirming the presence of NAD^+^ and MitoQH_2_ in NM NPs. The peaks around 1430, 1208, and 1060 cm^−1^ were assigned to aromatic ring vibrations, P–O/P–O–C stretching, and C–O stretching vibrations associated with the phosphate-ribose backbone of NAD^+^ and the ether/phenolic structures of MitoQH_2_. In addition, the bands at approximately 750 and 690 cm^−1^ were attributed to aromatic C–H out-of-plane bending vibrations, further supporting the incorporation of the triphenyl phosphonium-containing MitoQH_2_. Compared with free NAD^+^ and MitoQH_2_, the characteristic peaks of NM NPs exhibited slight broadening and shifts, suggesting intermolecular interactions between NAD^+^ and MitoQH_2_ during nanoparticle assembly. Furthermore, Tween 80 exhibited characteristic absorption bands at approximately 1735 cm^−1^, corresponding to ester C=O stretching vibrations, and around 1100 cm^−1^, attributed to ether C–O–C stretching vibrations. These characteristic Tween 80 peaks were not prominently detected in purified NM NPs, indicating efficient removal of Tween 80 during the purification process and minimal retention in the final formulation. Collectively, these results demonstrate the successful co-assembly of NAD^+^ and MitoQH_2_ and support the carrier-free nature of NM NPs. Compositional analysis via X-ray photoelectron spectroscopy (XPS) further confirmed the successful integration of both building blocks, with characteristic peaks for C1s, O1s, N1s, and P2p core levels corresponding to NAD^+^ and MitoQH_2_ (Fig. 1G and H).

The ultraviolet–visible (UV–Vis) absorption spectrum of NM NPs exhibited distinct features derived from both NAD^+^ and MitoQH_2_ (Fig. 1I). The characteristic peak of NAD^+^ around 260 nm originated from the π→π* electronic transition within its adenine ring system, while MitoQH_2_ showed a broad absorption band between 230 and 280 nm attributed to π→π* and n→π* transitions of the quinone structure. The spectrum of NM NPs displayed the combined features of both molecules, accompanied by a slight shift in peak position, suggesting molecular interactions within the assembled nanostructure. To gain deeper insight into the self-assembly mechanism and stability of NM NPs at the molecular level, molecular dynamics (MD) simulations were further performed. Representative MD snapshots captured at different simulation times (0–30 ns) illustrated the dynamic evolution of the system from a randomly dispersed state to a well-ordered, compact nanostructure (Fig. 1J). The evolution of intermolecular hydrogen bonds showed a rapid increase before plateauing (Fig. 1K), indicating the formation of a stable supramolecular network. In parallel, the solvent-accessible surface area (SASA) exhibited a rapid decrease within the first 10 ns and stabilized thereafter (Fig. 1L), reflecting the transition from a loose molecular assembly to a compact nanostructure. Moreover, Independent Gradient Model based on Hirshfeld partition (IGMH) analysis of representative conformations of NM NPs revealed isosurfaces dominated by green regions, indicating dispersion/hydrophobic and π–π contacts (Fig. S3), consistent with the proposed assembly drivers.

One of the key features of NM NPs is their ROS-triggered disassembly. When exposed to oxidative stress (e.g., superoxide anions), MitoQH_2_ underwent electron loss and was oxidized into MitoQ, resulting in structural destabilization of the nanostructure and consequent release of NAD^+^ and MitoQ molecules. To quantitatively evaluate this responsive behavior, we monitored the release kinetics of the encapsulated cargo under physiologically relevant oxidative stress conditions. As shown in Fig. 1M and N, NM NPs exhibited accelerated and sustained release of both NAD^+^ and MitoQ, whereas minimal release was observed in PBS, confirming the ROS-responsive properties of the formulation. In summary, we have successfully designed and characterized a novel, sub-15 nm nanoplatform that autonomously assembles from bioactive molecules without the need for synthetic polymers or covalent modification. NM NPs exhibit well-defined morphology, exceptional stability, and ROS-responsive disintegration and payload release. This design not only mimics natural supramolecular organization but also provides a robust and bioresponsive system for targeted delivery to oxidative stress-rich tissues such as the injured kidney, offering a promising therapeutic strategy for AKI.

### 2.2 Biodistribution and renal targeting of NM NPs

**Fig. 2:**
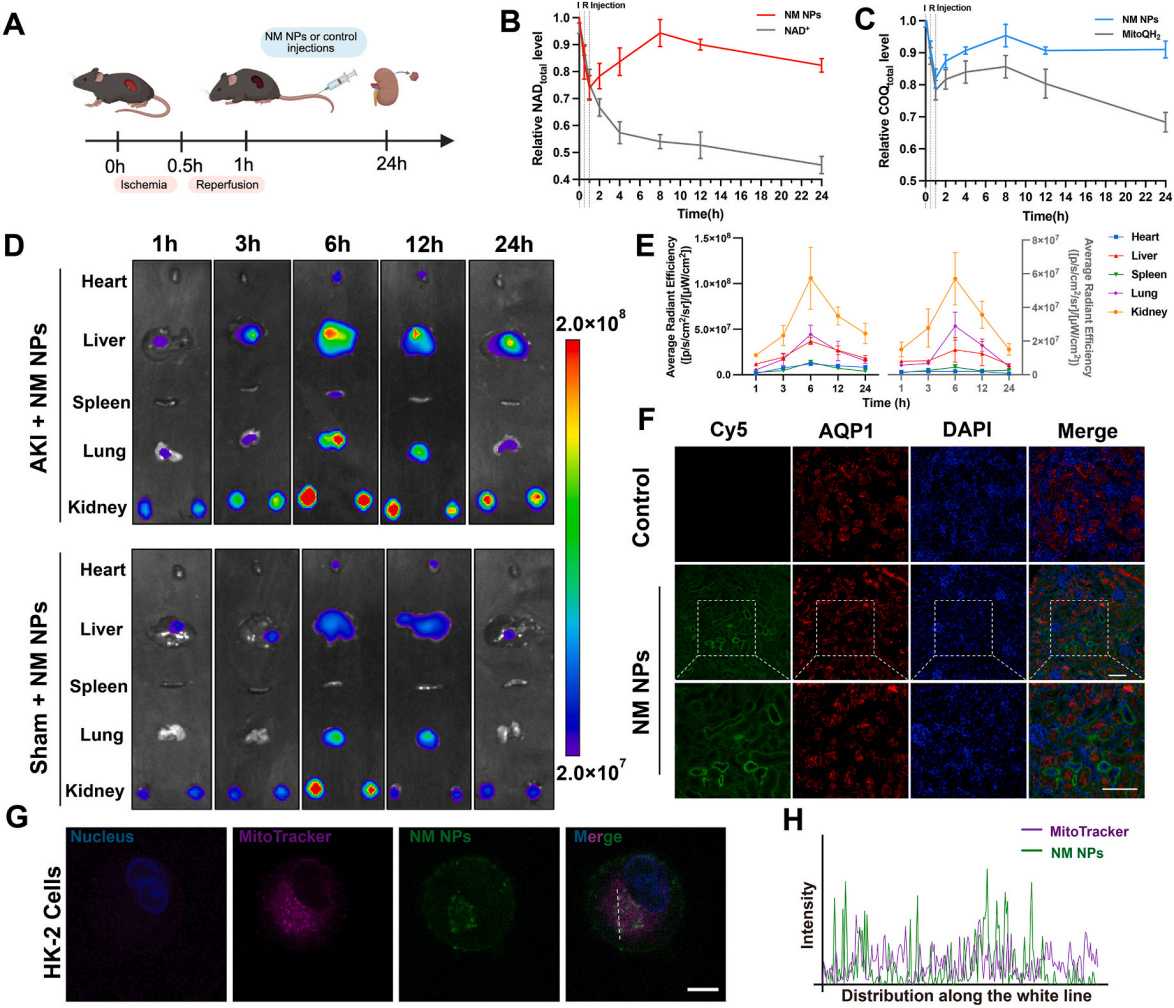
**Evaluation of the renal delivery efficiency of NM NPs.** (A) Schematic illustration of the experimental design. (B–C) Time-dependent changes in renal NAD_total_ and CoQ_total_ levels after IR-induced AKI and treatment with NM NPs or equivalent free molecules, n = 3 per group. (D) Ex vivo fluorescence imaging of major organs (heart, liver, spleen, lung, and kidney) collected at different time points after NM NP injection in AKI and sham groups. (E) Quantitative analysis of fluorescence intensities in major organs over time, n = 3 per group. (F) Confocal fluorescence images of kidney sections showing Cy5-labeled NM NPs (green), AQP1 (red, proximal tubular marker), and DAPI (blue). Scale bars = 50 μm. (G) Confocal microscopy of HK-2 cells showing colocalization of NM NPs with mitochondria stained by MitoTracker, indicating efficient mitochondrial targeting. Scale bars = 10 μm. (H) Line-scan analysis performed by ImageJ software along the white line. Data are presented as mean ± SD. (For interpretation of the references to colour in this figure legend, the reader is referred to the Web version of this article.)

Having established the ROS-responsive properties of NM NPs *in vitro*, we next sought to evaluate their biodistribution and renal targeting efficacy *in vivo*, a critical step for validating their therapeutic potential in AKI. We employed a murine model of bilateral renal IR injury. As outlined in Fig. 2A, AKI or sham-operated mice received a single injection of NM NPs (5 mg/kg) via intravenous administration at 0.5 h post-reperfusion. We first quantified the pharmacokinetics of the active components in kidney tissue. Following NM NPs administration, both NAD_total_ and CoQ_total_ levels in the kidney exhibited a rapid and sustained elevation, peaking within 8-10 h and remaining significantly above baseline for up to 24 h (Fig. 2B and C). In contrast, free NAD ^+^ failed to prevent the progressive decline of renal NAD_total_ after IR injury, likely due to its high hydrophilicity, poor membrane permeability, and limited intracellular bioavailability. Free MitoQH_2_ partially restored renal CoQ_total_ levels during the early phase, but this effect was less sustained than that observed with NM NPs. This pharmacodynamic profile confirms the efficient renal accumulation and prolonged retention of the released bioactive molecules, which is essential for combating the protracted oxidative stress in AKI. The superiority of the nano-formulation was further corroborated *in vitro*. Intracellular metabolite analysis at 6 h and 24 h revealed that, due to the high hydrophilicity and poor membrane permeability of NAD^+^, treatment with NAD ^+^ alone failed to elevate the NAD_total_ content in H/R-induced HK-2 cells (Figs. S4 and S5). In contrast, NM NPs were efficiently internalized and released NAD^+^ and CoQ in response to intracellular ROS, resulting in a marked increase in both NAD_total_ and CoQ_total_ levels. Moreover, NM NPs induced a greater elevation of intracellular CoQ compared with treatment with MitoQH_2_ alone, likely because the restoration of NAD^+^ by NM NPs rescued mitochondrial integrity and consequently promoted additional CoQ biosynthesis.

To visualize the spatiotemporal distribution pattern, we performed *ex vivo* fluorescence imaging using Cy5-labeled NM NPs. Quantitative analysis revealed a significantly higher renal accumulation in AKI mice compared to sham controls (Fig. 2D and E), underscoring a passive targeting mechanism leveraged by the pathological microenvironment. We attribute this enhanced retention in injured kidneys to two synergistic factors: impaired renal clearance function due to IR injury, and the transient enlargement of glomerular filtration pores during AKI, which facilitates the entrapment of our ultrasmall (∼10 nm) nanoparticles. Furthermore, the biodistribution profile showed altered accumulation in extra-renal organs. The increased fluorescence signals in the liver and lungs of AKI mice, compared to sham controls, are likely consequences of AKI-induced systemic pathology, including hepatic congestion and increased pulmonary vascular permeability [38]. The gradual decrease in fluorescence signals in major organs over time suggests progressive signal attenuation and possible clearance of Cy5-labeled NM NPs, indicating a relatively low tendency for short-term tissue retention.

To decipher the renal targeting at a cellular and subcellular resolution, we conducted detailed histological analyses. Immunofluorescence staining of kidney sections for aquaporin-1 (AQP1), a proximal tubular marker, demonstrated strong co-localization of Cy5 fluorescence with AQP1-positive tubules in NM NPs-treated AKI kidneys, a signal negligible in controls (Fig. 2F). These results support the preferential localization of NM NPs in AQP1-positive proximal tubules, which represent the primary site of injury in IR-AKI. Confocal microscopy in HK-2 cells further showed an apparent mitochondrial localization pattern of NM NPs (Fig. 2G), likely associated with the mitochondrial-targeting TPP ^+^ moiety of MitoQH_2_. Line-scan intensity analysis further supported the spatial overlap between the nanoparticle signal and the mitochondrial network (Fig. 2H).

The biosafety of NM NPs was then systematically validated. The biosafety of NM NPs was first evaluated after a single intravenous administration. Hematoxylin and eosin (H&E) staining of major organs, including the heart, liver, spleen, lung, and kidney, showed no noticeable structural abnormalities in NM NP–treated mice compared with the PBS group at 24 h post-injection (Fig. S6). Consistently, blood routine and serum biochemical analyses revealed no significant abnormalities among the treatment groups after single-dose administration (Fig. S7), suggesting good short-term systemic compatibility of NM NPs.

To further assess repeated-dose safety, mice were intravenously administered PBS, NAD^+^, MitoQH_2_, or NM NPs every 2 days from day 0 to day 10, followed by sample collection on day 12 (Fig. S8A). After completion of multiple treatment cycles, H&E staining of the heart, liver, spleen, lung, and kidney showed no obvious histopathological damage in the NM NP group (Fig. S8B). In addition, blood routine and serum biochemical parameters, including AST, ALT, Scr, BUN, RBC, WBC, LYM, HGB, and HCT, remained within comparable ranges among the different treatment groups (Fig. S9). These results indicate that NM NPs exhibit favorable systemic biosafety not only after single-dose administration but also after repeated intravenous exposure.

Together, these results demonstrate that NM NPs achieve selective renal accumulation in AKI, with preferential uptake by tubular epithelial cells and subsequent mitochondrial targeting, thereby supporting their potential for organ- and organelle-specific therapy in kidney injury, as well as a favorable biosafety profile and clinical translational potential. Notably, although NM NPs exhibited promising therapeutic efficacy and repeated-dose biosafety in the AKI mouse model, further studies are still needed before clinical translation. Future work should focus on long-term biodistribution and clearance, comprehensive immunogenicity and toxicity evaluation, pharmacokinetic optimization and large-animal validation.

### 2.3 In vitro therapeutic effects of NM NPs

**Fig. 3:**
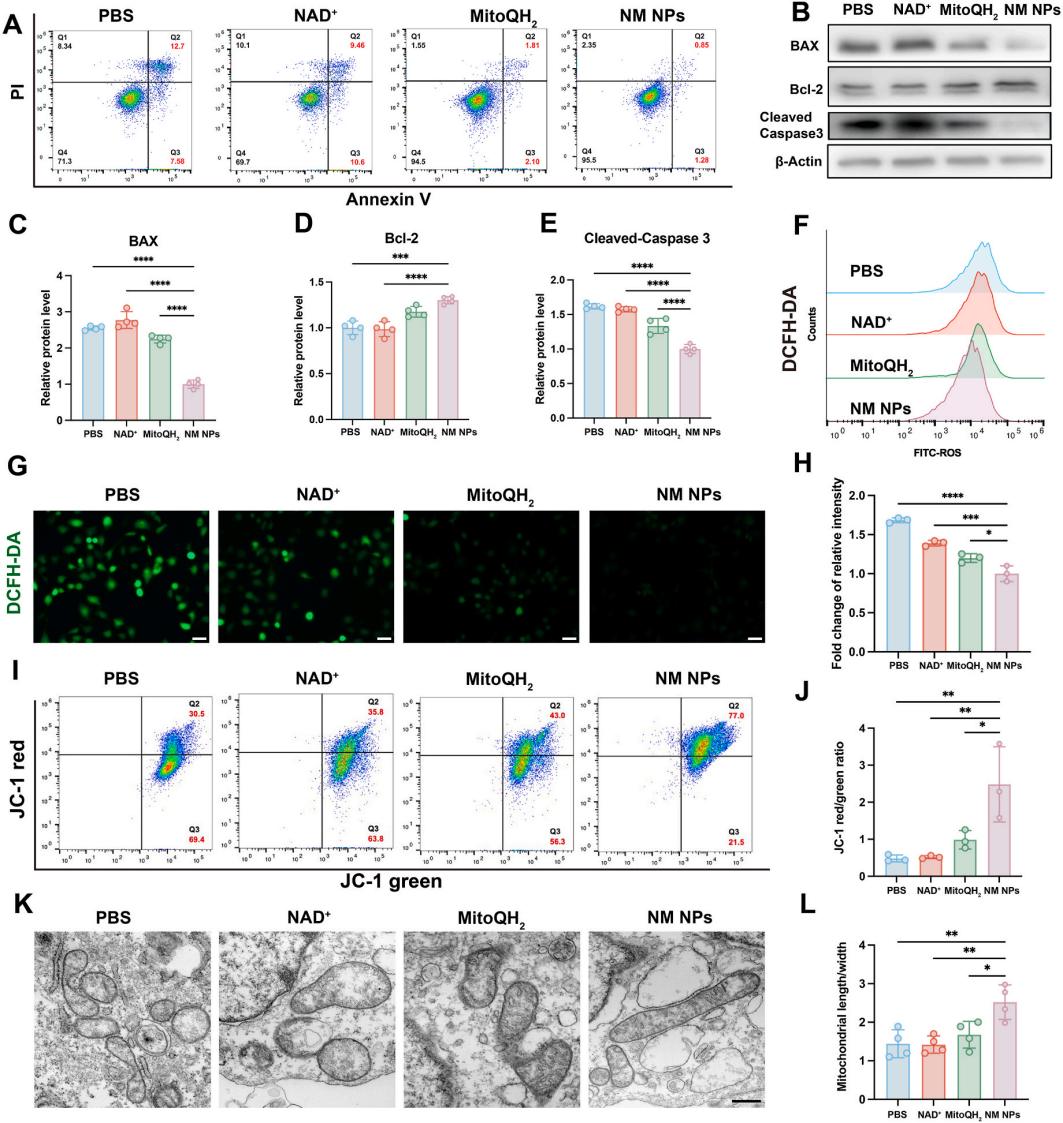
**In vitro therapeutic effect of NM NPs.** (A) Annexin V/PI flow cytometry of H/R-induced HK-2 cells across each group. (B-E) Western blot assays showing the expression and quantification of BAX, Bcl-2 and Cleaved Caspase 3 of H/R-induced HK-2 cells in each group, n = 4 per group. (F) Representative DCFH-DA flow histograms of intracellular ROS in each group. (G-H) Representative fluorescence images and fluorescence intensity quantification of DCFH-DA in H/R-induced HK-2 cells from each group, n = 3 per group. Scale bar = 50 μm. (I-J) Representative JC-1 flow cytometry and quantification of mitochondrial membrane potential in each group, n = 3 per group. (K-L) TEM images of mitochondrial ultrastructure and morphometric analysis in H/R-induced HK-2 cells from each group, n = 4 per group. Scale bar = 0.5 μm. Data are mean ± SD. P values by one-way ANOVA when comparing three or more groups * p < 0.05, **p < 0.01, ****p < 0.0001.

Building upon the demonstrated ROS-responsive release and targeted mitochondrial delivery, we next interrogated the cytoprotective efficacy of NM NPs in a cellular model of H/R- and cisplatin-induced injury. We hypothesized that the concerted action of NAD^+^ and MitoQ, delivered on-demand, would synergistically rescue mitochondrial integrity and bioenergetics, thereby averting apoptosis in HK-2 cells. The therapeutic superiority of NM NPs was first evident in their potent anti-apoptotic activity in both H/R- and cisplatin-induced HK-2 cell injury models. Flow cytometric analysis demonstrated that NM NP treatment markedly reduced the proportion of Annexin V^+^ apoptotic cells compared with the PBS, NAD^+^, or MitoQH_2_ groups (Fig. 3A, S10 and S11), indicating a pronounced anti-apoptotic effect. Western blot analysis further confirmed this finding (Fig. 3B–E), showing that NM NP treatment downregulated pro-apoptotic proteins BAX and Cleaved Caspase-3 while upregulating the anti-apoptotic protein Bcl-2. These results collectively indicated that NM NPs effectively attenuate apoptosis induced by oxidative stress. To further explore the antioxidant potential of NM NPs at the cellular level, we utilized the 2′, 7′-dichlorodihydrofluorescein diacetate (DCFH-DA) probe to detect intracellular ROS in H/R- and cisplatin-induced HK-2 cells (Fig. 3F–H and S12). Fluorescence imaging showed that H/R induced strong green fluorescence, confirming successful oxidative stress induction in HK-2 cells. Notably, NM NPs treatment significantly reduced fluorescence intensity, confirming that the nanoparticle not only delivers its payload but does so in a manner that effectively counteracts the oxidative burden.

Given the mitochondrion is both a primary source and a key target of ROS in AKI, we next performed a multi-faceted evaluation of mitochondrial health. JC-1 flow cytometry and fluorescence staining (Fig. 3I–J and S13) revealed that NM NPs substantially restored the mitochondrial membrane potential (ΔΨm), as indicated by an increased JC-1 red/green fluorescence ratio. Consistently, TEM showed that NM NP-treated cells maintained intact mitochondrial cristae and elongated morphology (Fig. 3K and L), whereas PBS and NAD ^+^ treated cells exhibited swollen mitochondria with disrupted membranes. While MitoQH_2_ mitigated oxidative stress to some extent in HK-2 cells under H/R-induced injury, its efficacy was consistently inferior to that of NM NPs. We attribute this enhancement to the synergistic provision of NAD^+^, which is essential for regenerating MitoQH_2_ from its oxidized form (MitoQ) within the mitochondrial electron transport chain, thereby amplifying the antioxidant cycle and sustaining its activity.

Collectively, these findings demonstrated that NM NPs conferred robust cytoprotection in renal tubular epithelial cells by suppressing ROS accumulation, preserving mitochondrial function, and preventing apoptosis. The synergistic release of NAD^+^ and MitoQ from ROS-responsive NM NPs provided simultaneous metabolic support and antioxidant defense, offering a promising therapeutic approach for mitochondrial dysfunction–associated renal injury.

### 2.4 NM NPs restore mitochondrial function by inhibiting ferroptosis and promoting autophagy

**Fig. 4:**
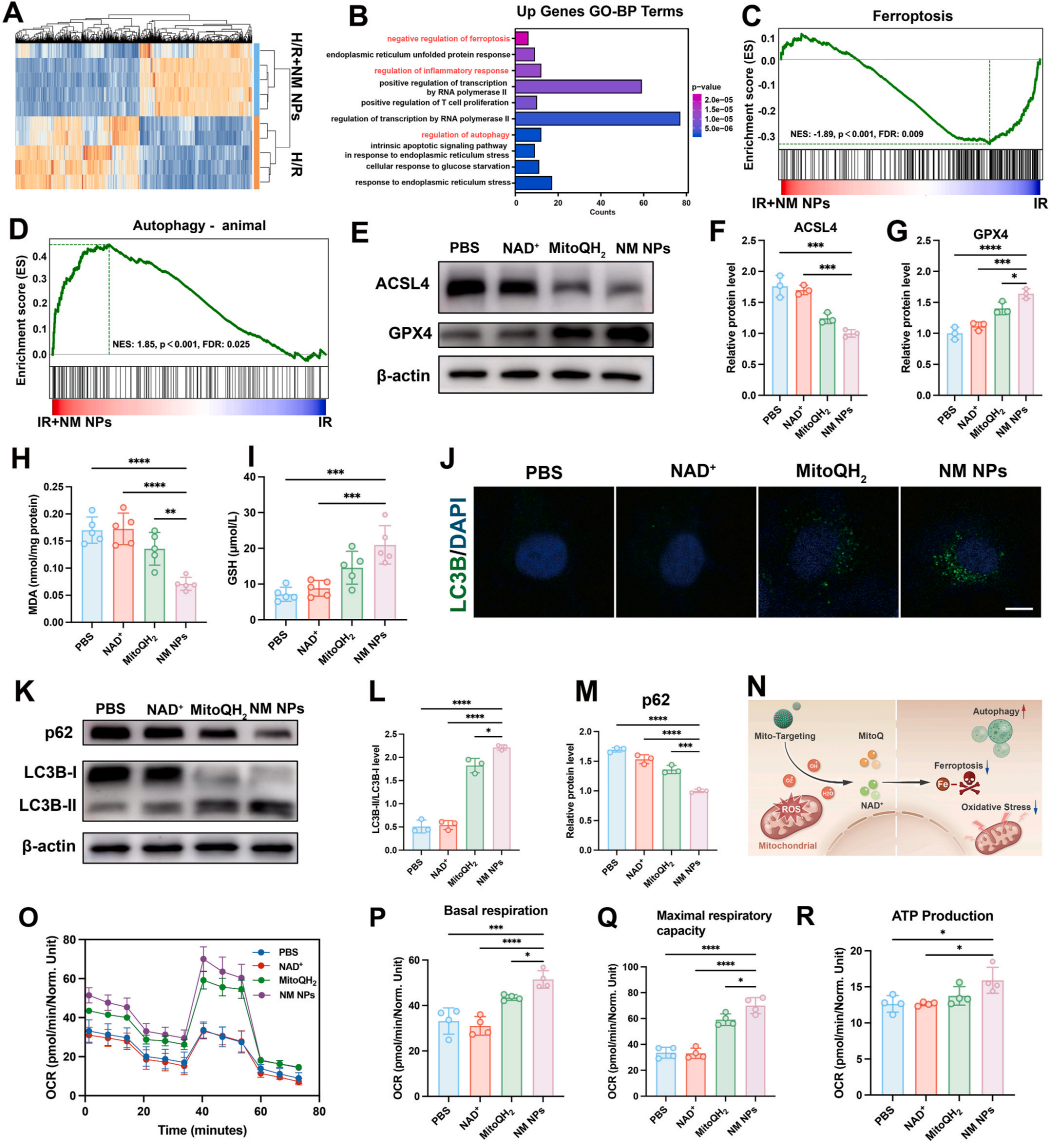
**Therapeutic mechanism of NM NPs in reversing AKI.** (A) Heatmap of differentially expressed genes between H/R and H/R + NM NPs in H/R-induced HK-2 cells, n = 5 per group. (B) GO-BP enrichment of genes upregulated by NM NPs, n = 5 per group. (C–D) GSEA of ferroptosis and autophagy gene set, n = 5 per group. (E-G) Western blot assays showing the expression and quantification of ACSL4 and GPX4 of H/R-induced HK-2 cells in each group, n = 3 per group. (H–I) Malondialdehyde and glutathione content of H/R-induced HK-2 cells in each group, n = 5 per group. (J) Representative fluorescence images of LC3B in H/R-induced HK-2 cells from each group. Scale bar = 10 μm. (K–M) Western blot assays showing the expression and quantification of p62 and LC3-II/I of H/R-induced HK-2 cells in each group, n = 3 per group. (N) Conceptual schematic of NM NPs suppressing ferroptosis and enhancing autophagy. (O-R) Quantification of basal respiration, maximal respiratory capacity, and ATP production from each group, n = 4 per group. Data are mean ± SD. P values by one-way ANOVA when comparing three or more groups * p < 0.05, **p < 0.01, ****p < 0.0001.

Having demonstrated the robust cytoprotective and bioenergetic effect of NM NPs against AKI, we next sought to elucidate the system-level molecular mechanisms underlying their therapeutic efficacy. Transcriptomic analyses indicated that NM NPs substantially reprogrammed the renal injury response in both H/R-induced HK-2 cells *in vitro* and the IR-AKI model *in vivo* (Fig. 4A, Fig. S14). Unsupervised clustering of differentially expressed genes clearly separated IR + NM NPs from the IR group, suggesting a profound impact of the nanotherapy on the global gene expression landscape in the injured kidney. Gene Ontology enrichment of upregulated genes in the IR + NM NPs group highlighted processes closely linked to regulated cell death and stress adaptation, including negative regulation of ferroptosis, regulation of autophagy, and inflammatory response terms (Fig. 4B). This suggested a strategic shift from uncontrolled cell death to managed stress adaptation. Consistent with these pathway-level trends, GSEA demonstrated a significant depletion of the ferroptosis signature in IR + NM NPs compared with IR (Fig. 4C), while the autophagy gene set was significantly enriched (Fig. 4D). Intriguingly, pathways directly related to the delivered bioactives, such as NAD^+^ binding and NAD^+^-dependent protein ADP-ribosyltransferase activity, were also significantly enriched (Fig. S15), linking the nanoparticle's composition directly to the observed transcriptomic rewiring.

To bridge this transcriptomic profile to functional pathophysiology, we validated the ferroptosis axis at the protein and metabolic levels. Western blotting showed that NM NPs reduced the expression of ACSL4, a key pro-ferroptotic enzyme that enriches membranes with peroxidation-prone polyunsaturated phospholipids, whereas GPX4, the central glutathione-dependent lipid hydroperoxidase that detoxifies lipid peroxides, was increased in the NM NPs group compared with PBS and free-drug controls (Fig. 4E–G). In line with these molecular changes, NM NPs significantly decreased renal MDA levels, indicating reduced lipid peroxidation, and restored GSH content, reflecting improved antioxidant capacity (Fig. 4H and I). These data provide a clear mechanistic link, from gene expression to enzyme activity to lipid peroxidation, demonstrating that NM NPs effectively shut down the ferroptosis execution pathway.

In parallel, we confirmed the activation of a pro-survival autophagy flux. Immunofluorescence staining of the autophagosome marker LC3B revealed a pronounced increase in punctate structures in the NM NPs group (Fig. 4J), indicative of enhanced autophagic activity. Western blotting analyses further demonstrated an increased LC3B-II/LC3B-I ratio and a concomitant reduction in p62 abundance relative to controls (Fig. 4K–M), supporting activation of autophagy and more efficient clearance of ubiquitinated cargo. Together, these data provide multi-level evidence that NM NPs attenuate IR-induced kidney injury by inhibiting ferroptosis (ACSL4↓, GPX4↑, MDA↓, GSH↑) while simultaneously promoting autophagy (LC3B puncta↑, LC3B-II/I↑, p62↓), suggesting a coordinated mechanism that limits oxidative membrane damage and enhances cellular stress tolerance (Fig. 4N). To further elucidate the functional consequences of these mitochondrial improvements, cellular oxygen consumption rate (OCR) was measured using a Seahorse extracellular flux analyzer. NM NP treatment significantly enhanced basal respiration, maximal respiratory capacity, and ATP production compared to the other groups (Fig. 4O–R). These results demonstrated that NM NPs not only prevent mitochondrial damage but also restore oxidative phosphorylation efficiency, thereby improving overall cellular energy metabolism.

### 2.5 NM NPs protected mice from renal ischemia‒reperfusion-induced AKI and inflammation

**Fig. 5:**
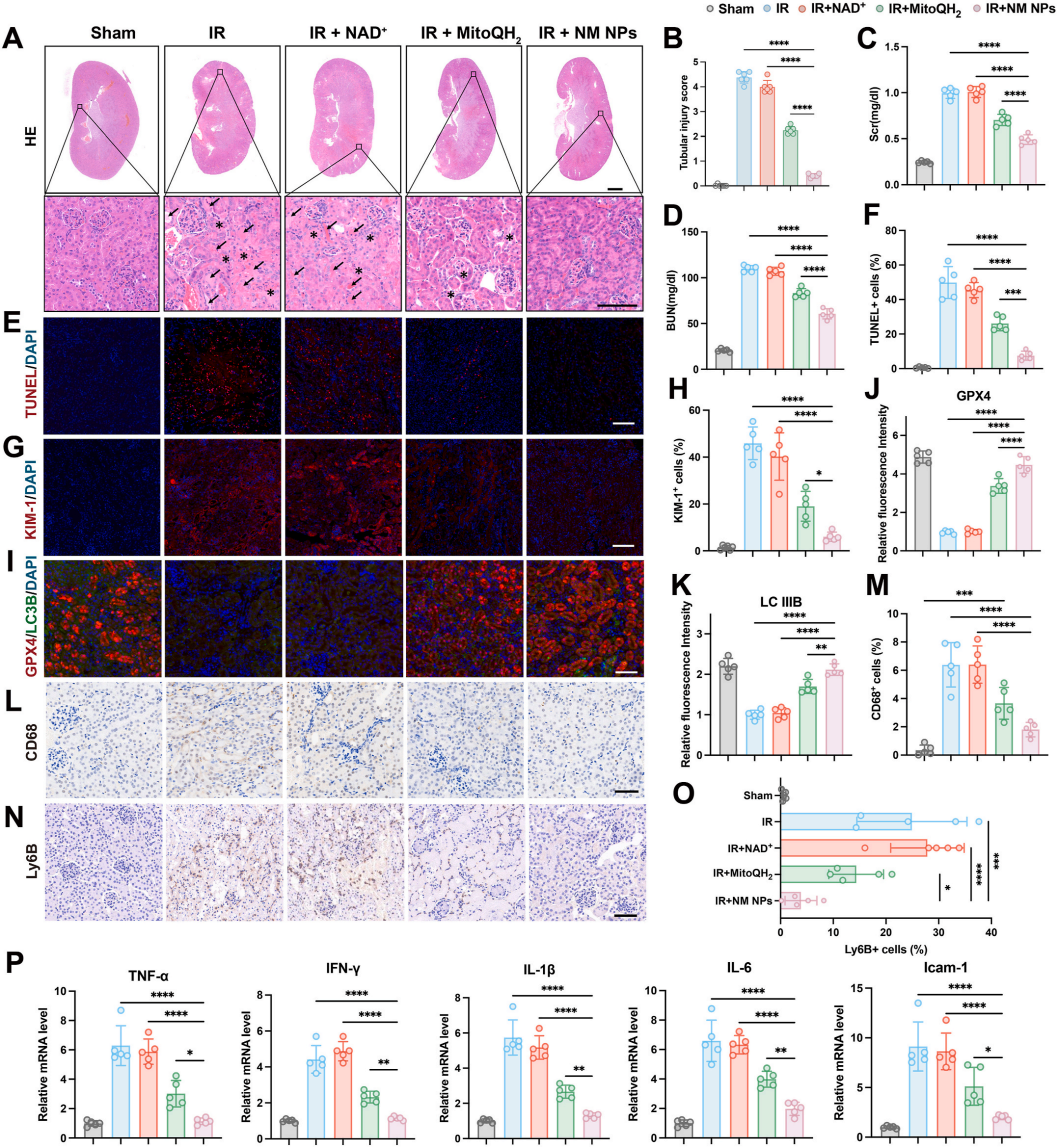
**NM NPs prevented renal IR-induced AKI and inflammation.** (A) H&E staining of kidneys from Sham, IR, IR+NAD^+^, IR+MitoQH_2_, and IR+NM NPs groups; Arrows indicate epithelial cell detachment, cellular debris accumulation, and cast formation, and asterisks represent atrophic, flattening epitheliums. Scale bars, upper panels = 1 mm; lower panels = 100 μm. (B) Tubular injury score assessed by the HE staining images in each group, n = 5 per group. (C–D) Serum creatinine (Scr) and blood urea nitrogen (BUN) in each group, n = 5 per group. (E-F) TUNEL staining and quantification of TUNEL^+^ cells of each group, n = 5 per group. Scale bar = 100 μm. (G–H) KIM-1 immunofluorescence and percentage of KIM-1^+^ cells of each group, n = 5 per group, Scale bar = 100 μm. (I–K) Representative immunofluorescence images and fluorescence intensity quantification of GPX4 and LC3B in each group, n = 5 per group. Scale bar = 75 μm. (L–M) Immunohistochemical images and quantification of CD68^+^ macrophages in each group, n = 5 per group. Scale bar = 25 μm. (N-O) Immunohistochemical images and quantification of Ly6B^+^ neutrophils in each group, n = 5 per group. Scale bar = 25 μm. (P) Relative mRNA levels of inflammatory mediators (TNF-α, IFN-γ, IL-1β, IL-6, Icam-1) in the kidney tissues in each group, n = 5 per group. Data are mean ± SD. P values by one-way ANOVA when comparing three or more groups * p < 0.05, **p < 0.01, ****p < 0.0001.

The *in vivo* therapeutic efficacy of ultrasmall NM NPs was then tested in a murine model of bilateral renal IR injury. Sham-operated mice were included as normal controls to define baseline renal function and renal histological morphology. Mice were subjected to renal IR and subsequently treated via tail vein injection with PBS, free NAD^+^, MitoQH_2_, and NM NPs, to determine whether the nanoscale assembly translates into superior functional and structural recovery. Examination of kidney histology provided immediate and compelling evidence of the therapeutic efficacy. HE and periodic acid–Schiff (PAS) staining revealed that bilateral renal IR injury caused severe morphological damage, including loss of the tubular brush border, tubular dilatation, accumulation of cellular debris, and cast formation (Fig. 5A and B and Fig. S16). These pathological changes were markedly attenuated in the NM NPs group. Morphological observation and tubular injury scores demonstrated that NM NPs effectively repaired the brush border, preserved renal tissue integrity, and alleviated renal damage compared with the other IR injury groups, indicating potent protection against IR-induced tubular injury. Critically, this structural preservation was directly linked to the restoration of kidney function. Biochemical analyses demonstrated that NM NPs treatment led to a striking reduction in both Scr and BUN levels at 24 h post-injury (Fig. 5C and D), indicating a rapid rescue of the glomerular filtration rate. This functional recovery was underpinned by a significant reduction in tubular cell death, as evidenced by diminished TUNEL staining (Fig. 5E and F). Furthermore, the expression of Kidney Injury Molecule-1 (KIM-1), a sensitive and specific biomarker for proximal tubular damage, was also markedly lowered by NM NPs treatment (Fig. 5G and H).

We then sought to validate, at the tissue level, the key mechanistic pathways identified by our transcriptomic analysis. Immunofluorescence on kidney sections revealed that NM NPs significantly enhanced GPX4 expression, reflecting suppression of lipid peroxidation, and simultaneously increased LC3B levels, suggesting activation of autophagy to maintain mitochondrial homeostasis (Fig. 5I–K). These coordinated effects indicated that NM NPs effectively relieved mitochondrial oxidative injury and promoted cellular adaptive repair, which was consistent with our *in vitro* findings and explained the profound cytoprotection observed. In addition to mitigating structural and metabolic damage, NM NPs also exerted potent anti-inflammatory effects. Immunohistochemical staining for CD68 and Ly6B revealed dense macrophage and neutrophil infiltration in the IR group, which was attenuated by NM NPs (Fig. 5L–O). Consistently, qRT-PCR analysis showed that several inflammatory genes (TNF-α, IFN-γ, IL-1β, IL-6 and Icam-1) were significantly downregulated in NM NP–treated kidneys compared with the IR and monotherapy groups (Fig. 5P).

### 2.6 Broad-spectrum renoprotection: NM NPs protected mice from renal cisplatin -induced AKI

**Fig. 6:**
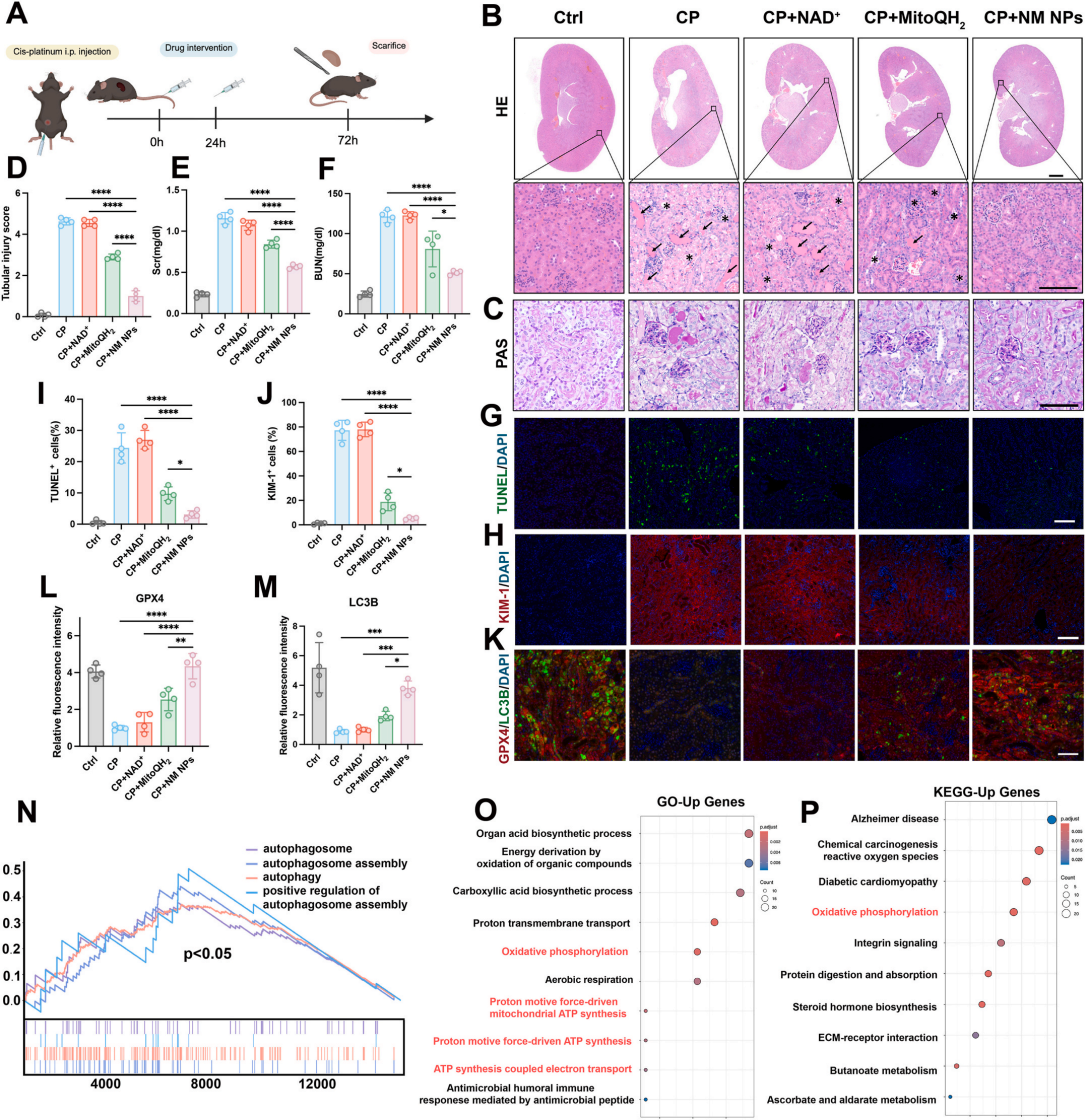
**NM NPs mitigate cisplatin-induced AKI and reprogram injury-associated transcriptional programs.** (A) Schematic of the *in vivo* study design. Acute kidney injury (AKI) was induced by intraperitoneal cisplatin injection. Mice received PBS, NAD^+^, MitoQH_2_, or NM NPs at 0 h and 24 h after cisplatin administration and were sacrificed at 72 h for sample collection. (B) Representative H&E-stained kidney sections. Arrows indicate epithelial cell detachment, cellular debris accumulation, and cast formation, and asterisks represent atrophic, flattening epitheliums. Scale bars, upper panels = 1 mm; lower panels = 100 μm. (C) Representative PAS-stained kidney sections assessing tubular structural alterations. Scale bar = 100 μm. (D-F) Tubular injury score, serum creatinine and blood urea nitrogen in each group, n = 4 per group. (G, I) TUNEL staining and quantification of TUNEL^+^ cells of each group, n = 4 per group. Scale bar = 100 μm. (H, J) KIM-1 immunofluorescence and percentage of KIM-1^+^ cells of each group, n = 4 per group. Scale bar = 100 μm. (K-M) Representative immunofluorescence images and fluorescence intensity quantification of GPX4 and LC3B in each group, n = 4 per group. Scale bar = 75 μm. (N) GSEA illustrating pathway-level changes in the autophagy-related gene sets following NM NPs treatment, n = 4 per group. (O-P) Bubble plots summarizing significantly enriched pathways (GO/KEGG) among regulated genes, n = 4 per group. Data are mean ± SD. P values by one-way ANOVA when comparing three or more groups * p < 0.05, **p < 0.01, ****p < 0.0001.

To evaluate the therapeutic versatility of our nanoplatform beyond IR injury, we investigated its efficacy in cisplatin-induced acute kidney injury (CP-AKI)—a clinically prevalent and mechanistically distinct nephropathy. Cisplatin possesses intrinsic nephrotoxic properties during cancer therapy, with renal injury largely driven by mitochondrial oxidative stress and ensuing mitochondrial dysfunction [39]. We hypothesized that the fundamental ability of NM NPs to restore mitochondrial homeostasis would confer protection across different AKI etiologies. We established a CP-AKI model by intraperitoneal administration of a high dose of cisplatin [40], with Ctrl mice included as normal controls without cisplatin challenge. Cisplatin-treated mice were administered PBS, NAD^+^, MitoQH_2_, or NM NPs at 0 h and 24 h after cisplatin challenge, and kidneys were harvested at 72 h (Fig. 6A). Histopathological examination revealed pronounced tubular damage in the PBS group, characterized by extensive epithelial injury and luminal casts, whereas NM NPs markedly alleviated these lesions and better-preserved tubular architecture on PAS staining (Fig. 6B and C). Consistently, NM NPs significantly lowered the tubular injury score and improved renal function, as reflected by reduced Scr and BUN compared with the CP group and the corresponding free-drug controls (Fig. 6D–F). We next quantified tubular cell death. TUNEL staining showed a substantial reduction in apoptotic cells in the NM NPs group (Fig. 6G and I). Immunofluorescence analysis also showed substantial reduction in KIM-1 expression (Fig. 6H and J), indicating effective mitigation of proximal tubular injury, the primary target of cisplatin toxicity. To determine whether the protective mechanisms identified in CP-AKI were conserved in this model, we performed transcriptomic profiling. Unsupervised clustering clearly distinguished NM NP-treated kidneys from cisplatin controls (Fig. S17). We further assessed ferroptosis- and autophagy-associated changes in renal tissues from the cisplatin-induced AKI model. Immunofluorescence staining showed that cisplatin injury markedly decreased GPX4 and LC3B expression, whereas NM NP treatment significantly restored both markers compared with CP and the free-component treatment groups (Fig. 6K–M). GSEA confirmed a significant enrichment of autophagy-related genes and depletion of ferroptosis signatures, recapitulating the core molecular pathways observed previously (Fig. 6N and S18). KEGG and GO enrichment analyses of upregulated DEGs consistently indicated enhanced oxidative phosphorylation and ATP synthesis in CP-AKI mice treated with NM NPs. Collectively, these results suggest that NM NPs mitigate cisplatin-induced mitochondrial oxidative stress and improve mitochondrial function (Fig. 6O and P). Together, these data support that NM NPs confer robust renoprotection in cisplatin AKI, providing a promising strategy to mitigate cisplatin-associated nephrotoxicity during cancer chemotherapy.

## 3 Conclusion

In summary, we have developed and comprehensively validated a minimalist, carrier-free nanomedicine, NM NPs, which integrates disease-microenvironment targeting with organelle-specific delivery to directly address mitochondrial dysfunction in acute kidney injury. This study provides a proof-of-concept strategy in nanotherapeutic design by demonstrating that the strategic co-assembly of two bioactive molecules, NAD^+^ and MitoQH_2_, can yield a therapeutic entity with emergent properties that surpass the simple sum of its parts.

This integrated design operates through a coherent, multi-level targeting strategy. The ultrasmall size (∼10 nm) enables passive renal targeting through the pathologically altered glomerular filtration barrier, achieving injury-selective accumulation. Upon entering proximal tubular epithelial cells, the ROS-responsive disassembly ensures spatiotemporally controlled release of the payload. The released NAD^+^ and MitoQH_2_ then function synergistically, not as independent agents but as complementary components of a self-reinforcing therapeutic cycle. While MitoQH_2_ directly neutralizes mitochondrial ROS to protect membrane integrity against ferroptosis, NAD^+^ replenishment concurrently fuels energy-dependent quality control processes like autophagy. This dual-pathway intervention collectively reprograms the cellular response to injury, ultimately restoring oxidative phosphorylation and rescuing bioenergetic capacity, which is indispensable for tissue repair.

The broad-spectrum efficacy of NM NPs, demonstrated in both ischemia-reperfusion and cisplatin-induced AKI models, underscores their potential as a versatile platform technology for mitochondrial rescue. The conserved protective effects observed in ischemia-reperfusion- and cisplatin-induced AKI models suggest that mitochondrial homeostasis may represent a shared therapeutic axis in these experimental settings. Together with the preliminary short-term and repeated-dose safety data, these findings support further preclinical evaluation of NM NPs as a mitochondria-targeted metabolic nanomedicine for AKI. It opens new avenues for organelle-specific medicine, not only for AKI but potentially for a wider class of pathologies rooted in mitochondrial dysfunction.

## 4 Experimental section

### 4.1 Ethics statement

The study adhered to the guidelines set by the Laboratory Animal Center of Shanghai General Hospital for the care and use of animals. All experimental protocols were reviewed and approved by the Animal Ethics Committee of Shanghai General Hospital (2022AW039).

### 4.2 Preparation of NM NPs

NAD^+^ (200 μg, MedChemExpress) and MitoQH_2_ (200 μg, MedChemExpress) were dissolved in 1 mL of deionized water, with MitoQH_2_ initially dissolved in DMSO. Tween 80 (10 μL, Meilunbio) was added, and the mixture was stirred overnight at 1500 rpm using a magnetic stirrer at 4 °C under light-protected conditions. The products were collected and dialyzed against water with repeated water changes to remove unreacted raw materials. For samples requiring concentration, the resulting suspension was then transferred to ultrafiltration tubes (molecular weight cut-off: 3.5 kDa) to remove unassembled free NAD^+^ and MitoQH_2_. Centrifugation was performed at 3000 rpm for 30 min at room temperature or 4 °C to obtain purified and concentrated NM NPs.

### 4.3 Characterization of NPs

The hydrated particle size, zeta potential, and stability of particles of NM NPs were measured by dynamic light scattering (DLS, Linkoptik). Transmission electron microscopy images of NM NPs were taken on an electron microscope (HT7700, Hitachi). The encapsulation efficiency and loading content of NAD^+^ and MitoQH_2_ in NM NPs were determined by LC-MS quantification. The filtrates collected during the ultrafiltration step of NM NP preparation were subsequently subjected to LC-MS analysis to quantify the amounts of unassembled free NAD^+^ and MitoQH_2_. The incorporated amounts of NAD^+^ and MitoQH_2_ were calculated based on the difference between the initial input amounts and the free components detected in the filtrates, and the encapsulation efficiency was determined accordingly. The purified NM NP suspension was then frozen in liquid nitrogen and lyophilized to obtain dry nanoparticle powders. The total mass of lyophilized NM NPs was accurately recorded, and the loading content of NAD^+^ and MitoQH_2_ was calculated based on the amount of each component incorporated into NM NPs relative to the total mass of the nanoparticles. FTIR was performed to characterize the molecular interactions involved in the formation of NM NPs. Briefly, purified NM NPs were lyophilized to obtain dry powders. Lyophilized NM NPs, NAD^+^, MitoQH_2_, and Tween 80 were analyzed using an FTIR spectrometer (Nicolet iS20, Thermo Scientific) equipped with an attenuated total reflectance (ATR) accessory. The spectra were recorded over the range of 4000–400 cm^−1^ at a resolution of 4 cm^−1^, with 32 scans collected for each sample. All spectra were background-corrected and normalized before comparison. Ultraviolet–visible absorption spectrum of NAD^+^, MitoQH_2_, and NM NPs were achieved by using UV–vis spectra (UV2700, Shimadzu). The elemental composition and valence were identified by X-ray photoelectron spectroscopy (XPS, Thermo Fisher). For molecular dynamics simulation, all molecules were parameterized using the GAFF force field, with topologies obtained from the AuToFF web server, and water molecules described by the OPC3 model. The simulation system contained MitoQH_2_, NAD^+^, and water molecules placed in a cubic box (5.65 × 5.65 × 5.65 nm^3^). Molecular dynamics simulations were conducted using the GROMACS package under periodic boundary conditions. A 2.0 fs integration step with the leapfrog algorithm was applied, and short-range van der Waals and electrostatic interactions were truncated at 1.4 nm. Long-range electrostatics were treated with the particle–mesh Ewald (PME) method (grid spacing 2 nm, interpolation order 4). Simulations were performed in the NPT ensemble at 298.15 K and 1 bar using the V-rescale thermostat and Berendsen barostat. The production run lasted 40 ns.

### 4.4 NAD^+^ and MitoQ release from NM NPs

NM NPs were immersed in two buffers (PBS, 0.1 mM H_2_O_2_) and incubated at 37 °C. Then, the supernatant of the NM NPs dispersion was collected and incubated for 1, 2, 4, 6, 12, and 24 h. The levels of NAD^+^ in each group were determined by NAD^+^/NADH detection kits (MedChemExpress). The levels of MitoQ in each group were determined by LC-MS.

### 4.5 In vivo distribution of NM NPs

Cy5-labeled NM NPs (5 mg/kg) were intravenously injected into healthy or ischemia-reperfusion-injured C57BL/6J mice. For *in vivo* imaging, mice were euthanized at 1, 3, 6, 12, and 24 h post-injection, and major organs including the heart, liver, spleen, lungs, and kidneys were harvested for fluorescence imaging using the IVIS Lumina imaging system to assess the distribution and intensity of Cy5 signals. For fluorescence images of tissue slices, AQP1 antibody was used to label proximal tubules, and DAPI was used to stain cell nuclei.

### 4.6 Colocalization of mitochondria and NM NPs

NM NPs (15 μg/mL) were stirred with FITC overnight at 300 rpm using a magnetic stirrer. The FITC-labeled NM NPs were then added to the DMEM/F12K culture medium of HK-2 cells and incubated for 6 h. Live-cell staining was performed using Hoechst for nuclei according to the protocols provided by Beyotime. Mitochondria were stained with MitoTracker Deep Red (Thermo Fisher Scientific) following the manufacturer's instructions. Fluorescent images were acquired using a confocal laser scanning microscope.

### 4.7 Injury and treatment of HK-2 cells

HK-2 cells were cultured in DMEM/F12K medium (Gibco) supplemented with 10% fetal bovine serum (FBS, Gibco) and 1% penicillin/streptomycin (p/s, Invitrogen). To mimic IR injury *in vitro*, an H/R (hypoxia/reoxygenation) model was established. Briefly, when HK-2 cells reached 80-90% confluency, the standard medium was replaced with serum-free medium, and cells were placed in a hypoxic incubator (1% O_2_, 5% CO_2_, 37 °C) for 12 h. Following the hypoxic challenge, cells were returned to normoxic conditions (21% O_2_, 5% CO_2_, 37 °C) for 4 to 6 h to initiate reoxygenation. During this reoxygenation phase, cells were treated with either PBS, NAD^+^, MitoQH_2_, or NM NPs (100 μg mL^−1^), where the NAD^+^ and MitoQH_2_ groups received equivalent molar doses corresponding to the NM NP formulation. To simulate cisplatin-induced renal injury *in vitro*, cells were exposed to cisplatin at a final concentration of 20 μM for 12 h. During cisplatin stimulation, cells were treated with either PBS, NAD^+^, MitoQH_2_, or NM NPs (100 μg mL^−1^), where the NAD^+^ and MitoQH_2_ groups received equivalent molar doses corresponding to the NM NP formulation. Mitochondrial damage of HK-2 cells was detected with ROS Assay Kit (Beyotime) and Mitochondrial membrane potential assay kit with JC-1 (Beyotime). Cellular NAD^+^/NADH levels were detected by kits (MCE, China) and CoQ levels were detected by kits (Yuanye Bio-Technology).

### 4.8 Flow cytometry analysis of HK-2 cells

The death of HK-2 cells was detected using Pharmingen PE Annexin V Apoptosis Detection Kit I (Beyotime). The mitochondrial membrane potential of HK-2 cells was detected by Mitochondrial membrane potential assay kit with JC-1 (Beyotime).

### 4.9 Seahorse assay

The cells were seeded in a seahorse XF cell culture microplate and incubated until reaching the desired cell density. Subsequently, the microplate was detected using a Seahorse XFe Analyzer (Agilent) to measure the O_2_ consumption rate (OCR). The experimental process strictly followed the user guide provided by Agilent.

### 4.10 Transcriptomics analysis

Total RNA from the collected HK-2 cells and kidney tissues was extracted using TRIzol reagent (Invitrogen) and subjected to mRNA-sequencing and data analysis by LC-Bio Technology Co., LTD. (Hangzhou, China).

### 4.11 Western blotting

Cells and kidney tissues were lysed by RIPA buffer, and protein concentrations were measured using a BCA kit. Then, the loading buffer was added, and the samples were denatured at 100 °C for 10 min. Proteins were then separated by SDS-PAGE and transferred onto PVDF membranes. The membranes were blocked with 5% skim milk at room temperature for 2 h. The membranes were incubated with the appropriate primary antibodies overnight at 4 °C, then with the secondary antibodies at room temperature for 2 h. Protein bands were visualized by a chemiluminescence detection system (Tanon 5200).

### 4.12 Immunofluorescence

The cells were fixed with 4% paraformaldehyde for 15 min and permeabilized with 0.5% Triton X-100 for 30 min at room temperature. The paraffin-embedded kidney tissues were dewaxed by xylene and rehydrated through a graded alcohol series. Then, both cells and slices were blocked with 5% goat serum for 30 min, followed by overnight incubation with primary antibodies. Next, the fluorescently labeled secondary antibodies were used, and the samples were stained with DAPI (10 μg mL^−1^) for 10 min. CLSM was applied to capture the fluorescent images.

### 4.13 AKI mice models

For the IR-induced AKI model, mice were anesthetized with sodium pentobarbital, and dorsal skin incisions were made to expose the kidneys. The renal pedicles were clamped to induce ischemia, during which rectal temperature was maintained and the kidneys were kept moist. After 30 min, the clamps were removed to allow reperfusion. Thirty minutes later, IR-injured C57BL/6J mice were intravenously treated with PBS, free NAD^+^, free MitoQH_2_, or NM NPs. The dose of NM NPs was 5 mg/kg, defined as the total amount of active ingredients, including NAD^+^ and MitoQH_2_, while the free NAD^+^ and free MitoQH_2_ groups were given at molar-equivalent doses to the corresponding components in NM NPs. After 24 h, the mice were euthanized, and blood and kidney samples were collected for kidney function evaluation, histological analysis.

For the cisplatin-induced AKI model, a single intraperitoneal injection of cisplatin (20 mg/kg, Aladdin) was administered. Following cisplatin injection, mice in each group received PBS, NAD^+^, MitoQH_2_, or NM NPs once daily for two consecutive days. After 72 h, the mice were euthanized, and blood and kidney samples were collected for kidney function evaluation, histological analysis.

### 4.14 Blood analysis

Blood was collected from the orbital in living C57BL/6 mice under sodium pentobarbital. The collected blood samples were centrifuged for 20 min at 3500 rpm. The Scr and BUN were determined using commercial kits according to the manufacturer's protocol.

### 4.15 Histological examination

Kidney tissues from each group were fixed with 4% paraformaldehyde and embedded in paraffin. Then, cross-section slices were obtained for histological examination. H&E staining was performed according to the manufacturer's instructions (Solarbio). At least five fields per sample were imaged using an Olympus VS200 microscope.

### 4.16 Statistical analysis

The significance of the differences in mean values between and within multiple groups was determined by one-way ANOVA followed by Tukey's multiple range test when the data were normally distributed and were determined by the Kruskal–Wallis test when the data were abnormally distributed. Sample size (n) for each statistical analysis was shown in figure legends. p < 0.05 was considered statistically significant.

## Funding

This work was financially supported by the 10.13039/501100001809National Natural Science Foundation of China (82373178, 82470705).

## CRediT authorship contribution statement

**Huaze Ding:** Conceptualization, Data curation, Investigation, Methodology, Visualization, Writing – original draft. **Xinhui Huang:** Conceptualization, Data curation, Investigation, Methodology, Validation, Writing – original draft. **Peng Hu:** Conceptualization, Data curation, Investigation, Methodology. **Xiaoyan Zhang:** Conceptualization, Data curation, Formal analysis, Funding acquisition. **Yulin Wang:** Investigation, Methodology, Project administration. **Baiwei Mao:** Data curation, Formal analysis. **Jianxin Ye:** Methodology, Project administration. **Dianwen Song:** Formal analysis, Funding acquisition. **Chong Zhang:** Funding acquisition, Investigation, Software, Supervision. **Changping Wang:** Funding acquisition, Investigation, Methodology, Validation, Visualization, Writing – original draft.

## Declaration of competing interest

The authors declare that they have no known competing financial interests or personal relationships that could have appeared to influence the work reported in this paper.

## Data Availability

Data will be made available on request.
