# Supplementary material for: Rewiring the mitochondrial NAD+–CoQ axis using metabolic nanomedicine for acute kidney injury therapy
Source: Mater Today Bio. 2026 Jul 22;39:103479. doi: 10.1016/j.mtbio.2026.103479 (PMC13449347; doi:10.1016/j.mtbio.2026.103479)
Supplement: Supplementary file 1 [file mmc1.docx]

**Figure S1**. TEM image of NM NPs after storage at 4 °C for 72 h. Scale bar = 10 nm.

**Figure S2**. Time-dependent changes in the PDI of NM NPs over 72 h, n = 3 per group.

**Figure S3**. IGMH visualization of noncovalent interactions involved in the formation of NM NPs. (A) Left: δg–sign(λ₂)ρ scatter (IGMH, δg^inter; iso = 0.015 a.u.; color range −0.05–0.05 a.u.), where blue/green/red denote attractive, dispersion-dominated, and repulsive regions, respectively. (B) δg^inter isosurfaces for two representative conformations under identical settings. NAD⁺ and MitoQH_2_ show predominantly green patches.

**Figure S4.** Relative levels of NAD_total_ and CoQ_total_ in HK-2 cells after various treatments at 6 h, normalized to the control group, n = 5 per group.

**Figure S5.** Relative levels of NAD_total_ and CoQ_total_ in HK-2 cells after various treatments at 24 h, normalized to the control group, n = 5 per group.

**Figure S6.** H&E-stained sections of major organs (heart, liver, spleen, lung, kidney) collected from mice 24 h after intravenous administration of various groups. Scale bar = 100 μm.

**Figure S7.** Blood routine and biochemical analysis of various treatment groups after intravenous administration for 24 h, n = 5 per group.

**Figure S8.** (A) Schematic diagram of the repeated-treatment protocol for evaluating in vivo repeated-dose biosafety. (B) H&E-stained sections of major organs (heart, liver, spleen, lung, kidney) collected from mice after completion of repeated treatments in the indicated groups. Scale bar = 100 μm.

**Figure S9.** Blood routine and biochemical analysis of various treatment groups after repeated intravenous administration, n = 4 per group.

**Figure S10.** Apoptosis rate of H/R-induecd HK-2 cells across each group, n=3 per group.

**Figure S11.** Annexin V/PI flow cytometry of cisplatin-induced HK-2 cells across each group, n=4 per group.

**Figure S12.** Representative DCFH-DA flow histograms of intracellular ROS of cisplatin-induced HK-2 cells in each group.

**Figure S13.** Representative fluorescence images of JC-1 in H/R -induced HK-2 cells from each group. Scale bar = 50 μm.

**Figure S14.** Heatmap of differentially expressed genes between IR and IR+NM NPs in IR-AKI mice model.

**Figure S15.** GSEA of ferroptosis, autophagy, and NAD⁺-related gene sets in the IR-AKI mouse model.

**Figure S16.** Representative PAS-stained kidney sections assessing tubular structural alterations in IR-induced AKI model. Scale bar = 100 µm.

**Figure S17.** Heatmap of differentially expressed genes between CP and CP+NM NPs in CP-AKI mice model.

**Figure S18.** GSEA illustrating pathway-level changes in the ferroptosis-related gene set following NM NPs treatment, n = 4 per group.

| NAD^+^ | 87.39 ± 1.61 % |
| --- | --- |
| MitoQH_2_ | 69.31 ± 1.38 % |

**Table S1.** Encapsulation efficiency of NAD^+^ and MitoQH_2_ into NM NPs. Mean ± SD, n = 5 per group.

| Component | Drug loading content (%) | Loading amount (μg/mg NM NPs) |
| --- | --- | --- |
| NAD^+^ | 52.48 ± 1.18 | 524.8 ± 11.8 |
| MitoQH_2_ | 41.54 ± 0.85 | 415.4 ± 8.5 |
| Total | 94.02 ± 1.47 | 940.2 ± 14.7 |

**Table S2.** Drug loading content of NAD⁺ and MitoQH₂ in NM NPs. Mean ± SD, n = 5 per group.
